# Transmission of Dolutegravir resistance in treatment-naive individuals with HIV-1: A cohort study

**DOI:** 10.1016/j.bjid.2025.104513

**Published:** 2025-02-12

**Authors:** Jorge Francisco da Cunha Pinto, Luiza Brito Gomes, Natalia Dias Melo, Fabiana Barbosa Assumpção de Souza, Debora Viana Freitas, Sara Gonzalez Viega, Erica Ramos dos Santos Nascimento, Lidia Theodoro Boullosa, Cynthia Chester Cardoso, Amilcar Tanuri

**Affiliations:** a Universidade Federal do Estado do Rio de Janeiro (UNIRIO), Hospital Universitário Gaffrée e Guinle, Rio de Janeiro, RJ, Brazil; b Universidade Federal do Rio de Janeiro (UFRJ), Departamento de Genética, Laboratório de Virologia Molecular, Rio de Janeiro, RJ, Brazil

**Keywords:** Dolutegravir, Drug resistance, Antiretroviral therapy, HIV-1

## Abstract

**Background:**

Dolutegravir (DTG) is widely used as a first-line Antiretroviral Therapy (ART) due to its high efficacy and safety. However, concerns about DTG resistance persist. This study investigated the prevalence and factors associated with transmitted DTG resistance in treatment-naive HIV-1-infected individuals in Brazil.

**Methods:**

The study followed 150 treatment-naive HIV-1 individuals from May 2019 to May 2022 at a reference center for HIV/AIDS in Rio de Janeiro, Brazil. Baseline characteristics, viral load, and CD4 + cell counts were assessed. Genotypic resistance testing was conducted on plasma samples at baseline, and viral load was monitored during follow-up visits.

**Results:**

One hundred and thirty-one patients completed the study. The mean age was 37.73-years; 107 were male, and 24 were female. The median baseline of viral load was 4.33 log (21,193 copies/mm^3^), and CD4 + count was 342 cells/mm^3^, with the lowest count being 8 cells/mm^3^. The mean CD4 + count increase was 112 cells/mm^3^ (*p* < 0.01). One hundred and nine patients achieved an undetectable viral load three months after starting ART, with only eight patients not reaching undetectable levels by six months (42‒106 copies/mm^3^). The most common early adverse effect was nausea (12.9 %), and the most common later effect was increased creatinine levels (9.1 %), leading to the suspension or substitution of Tenofovir Disoproxil Fumarate (TDF). Genotyping was successfully performed on 85 patients: 66 were subtype B, 9 subtype C, 8 subtype F, and two CRF47_BF, with no resistance mutations and one accessory mutation (T97A).

**Conclusion:**

This study did not demonstrate transmitted DTG resistance among treatment-naive HIV-1-infected individuals. The findings suggest that DTG remains a safe and effective first-line ART option. However, close monitoring of viral load is recommended for all patients on DTG-containing ART regimens. Additionally, genotypic resistance testing should be performed on individuals who experience virological failure or a significant decline in CD4 + cell counts, with close attention to ART adherence.

## Introduction

Since January 2017, the Department of Chronic Conditions and Sexually Transmitted Infections of the Ministry of Health (MS) has started offering Dolutegravir (DTG) to patients who have developed resistance to previous medications, as well as for first-line treatment due to its higher efficacy and fewer adverse effects. The implementation of this treatment has once again positioned Brazil as a leading figure in the global response to HIV/AIDS. The recommended treatment regimen by the MS for initial treatment consists of a combination of reverse transcriptase inhibitors, specifically TDF and lamivudine (3TC), in conjunction with the integrase inhibitor DTG.^1^

To date, literature data indicates that DTG presents a higher genetic barrier to resistance compared to other Integrase Strand Transfer Inhibitors (INSTIs) and has shown to be safe for patients in randomized clinical trials.^2^, ^3^, ^4^ Results indicate that 88‒90 % achieve undetectable viral load after 48-weeks of treatment. Thus far, few cases of resistance have been reported with initial DTG-containing regimens, and the incidence of adverse effects has also been reduced in clinical trials.^3^ Currently, in Brazil, approximately 76,713 individuals are using DTG as first-line treatment, and another 45,645 have switched to DTG. In total, over 122,000 Brazilians living with HIV are utilizing DTG in their antiretroviral treatment regimens, representing 19 % of the total of 572,000 Brazilians receiving free antiretroviral treatment through the SUS (Unified Health System). It is significant that 87 % of individuals who initiated treatment in 2018 started with DTG.^5^

Data primarily come from international studies, mostly controlled trials. There is still a lack of data from Brazil to confirm whether resistance to DTG is indeed rare in our country. Considering that Brazil has adopted the initiative to administer DTG-containing regimens to all newly diagnosed patients, it becomes necessary to understand the profiles of primary resistance to this medication in our population and monitor the emergence time of mutations to this integrase inhibitor.

We developed a longitudinal study that simultaneously characterizes viral and human genetic variability will allow for an integrated approach to addressing the issue of resistance.

## Material and methods

### Study design and target population

This observational and prospective study targeted newly diagnosed HIV cases referred by Anonymous Testing Centers (ATCs) or, during the COVID-19 pandemic, through direct access. These individuals subsequently initiated clinical follow-up at the Hospital Universitário Gaffrée e Guinle (HUGG). Eligible participants were HIV-positive adults aged 18-years or older, of any sex, with no prior history of antiretroviral treatment.

We conducted a study with a convenience sample of 150 individuals, with a minimum follow-up period of up to 24-months, aiming to identify both primary resistance and the emergence of resistance during treatment with Brazil's first-line antiretroviral drugs. All necessary clinical and demographic information for the study was obtained from the review of each participant's medical records.

The identification of eligible individuals and their inclusion in the study was determined by the physician from the scientific support team. We collected viral load measurements at the initial visit, after three months of treatment, and subsequently every six months. All participants with sufficient viral load underwent genotyping to assess primary resistance profiles of HIV to DTG. Throughout the follow-up period, routine clinical monitoring of patients was conducted, including medical consultations and CD4 + cell count testing, when relevant. After the first viral load collection, if a sequential sample with detectable viral load above 1000 copies/mL was identified, a new genotyping analysis was performed to identify any resistance to the inhibitors present in the current therapeutic regimen.

### Strategy for sample and clinical data collection

At the time of participant inclusion in the study, the remaining material from the viral load test was sent to the Molecular Virology laboratory at Universidade Federal do Rio de Janeiro (UFRJ), where it was stored until the time of analysis. Similarly, the remaining material from all viral load tests performed by the participant during the 24-month follow-up period was also sent to the laboratory. The clinical follow-up of the participants was not altered and followed the natural course of outpatient care at the facility. All necessary information for the project was obtained from the medical records.

### HIV genotyping

Genotyping for monitoring antiretroviral resistance was performed using an in-house methodology developed and validated in a previous study by the Molecular Virology Laboratory at UFRJ and the WHO.^6^^,^^7^ The HIV genotyping assays aim to sequence the pol gene, amplifying the major regions of therapeutic interest of HIV-1 (Protease, Reverse Transcriptase, and Integrase) through PCR amplification, followed by Sanger sequencing. Resistance profiles to antiretrovirals were determined for all patients with viral load above 1000 copies/mL. The identification of HIV resistance mutations was based on the list of mutations from the IAS-USA and the Stanford HIV Drug Resistance Database interpretation algorithm (https://hivdb.stanford.edu/hivdb/by-mutations). The Stanford CPR interpretation algorithm (http://cor.stanford.edu/cpr.cgi) was used for the analysis of primary resistance.

### Statistical analysis

Descriptive statistics were used to summarize the demographic and baseline clinical characteristics of the participants. Age was presented as the mean ± standard deviation, while categorical variables, including gender and HIV subtype, were reported as frequencies and percentages. Viral load and CD4 + cell counts were described using the median and interquartile range. To assess the changes in CD4 + count and viral load over time, paired Wilcoxon signed-rank test was applied; p-value of <0.05 was considered statistically significant.

The incidence of adverse effects was calculated as the percentage of the total population experiencing each specific effect.

Genotype frequencies were analyzed to determine the distribution of HIV subtypes and the presence of any resistance or accessory mutations.

All statistical analyses were conducted using R: A Language and Environment for Statistical Computing.^8^

### Ethics consideration

This study was conducted in accordance with the ethical principles outlined in the Declaration of Helsinki and was approved by the institutional Research Ethics Committee under the registration number CAAE 04789018.7.0000.5257. All participants provided informed consent prior to enrollment, ensuring their voluntary participation and understanding of the study's objectives, procedures, and potential risks. Confidentiality and anonymity of the participants were strictly maintained throughout the research process.

## Results

**Fig. 1:**
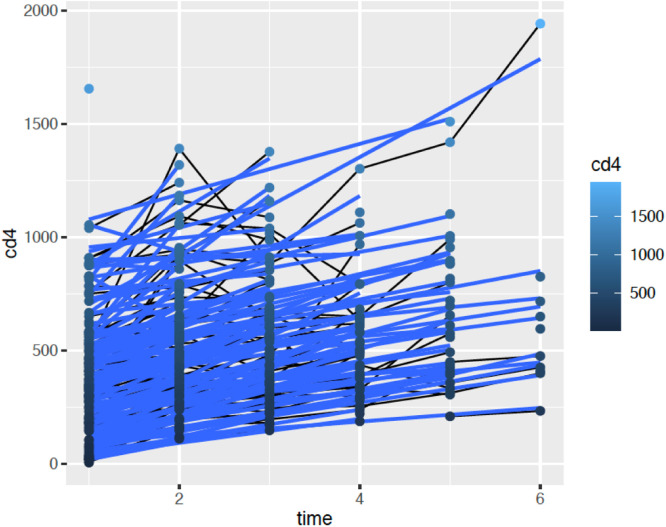
Time evolution of CD4 + count.

This study prospectively followed 150 treatment-naive HIV-1 individuals from May 2019 to May 2022, all of whom initiated the main antiretroviral regimen currently recommended in Brazil, consisting of a fixed-dose combination of Lamivudine 300 mg, Tenofovir Disoproxil Fumarate 300 mg, and Dolutegravir 50 mg. Of these, 131 participants completed the study. One patient succumbed to COVID-19, while 19 were lost to follow-up due to non-adherence to scheduled sample collection and clinic visits. Considering the patients who completed the study, 107 were male and 24 were female. The mean age was 37.73 ± 12.23 years. The minimum baseline CD4 + count was 8.0 cells/mm^3^, the median was 342 cells/mm^3^ and the maximum was 1319 cells/mm^3^. The patient with the lowest CD4 + count presented with a clinical picture of pneumocystosis and was successfully treated. The mean increase in CD4 + count was 112 cells/mm^3^ in six months (*p* < 0.01). Fig. 1 illustrates the time evolution of the CD4 + count. The mean increase in CD4 + count was 112 cells/mm^3^ in six months (*p* < 0.01). Fig. 1 illustrates the time evolution of the CD4 + count.

**Fig. 2:**
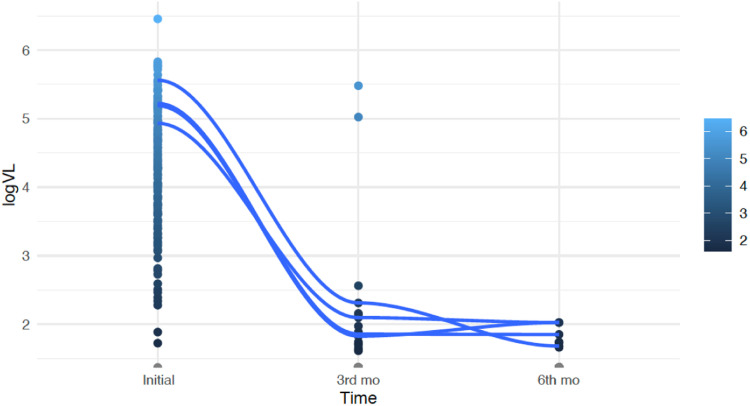
Viral load/Time.

The median viral load at the beginning of the study was 21,193 copies/mm^3^ (4.33 log). After three months of treatment, there was a significant reduction, with 84 % of the patients achieving undetectable viral load levels (*p* < 0.01). Two patients maintained high viral loads due to adherence difficulties. These patients showed a significant reduction in viral load measured at the sixth month of treatment. Overall, the mean reduction in viral load from baseline to the sixth month of treatment was 4.91 log, even when considering these two patients (*p* < 0.01). Fig. 2 illustrates the evolution of viral load over time.

The most common early adverse effect was nausea (12.9 %), and the most common later effect was an increase in creatinine levels (9.1 %), which led to the suspension or substitution of TDF, known to be associated with renal impairment. This action coincided with the new clinical management protocol for HIV infection from the Ministry of Health, which recommended dual therapy with 3TC and DTG.^9^ We successfully performed genotyping on 85 patients, while it was not possible to genotypically assess 46 individuals due to low viral load or amplification failure. Sixty-six were of subtype B, nine of subtype C, eight of subtype F, and two of CRF47_BF. None presented resistance mutations, and only one presented an accessory mutation (T97A). There were no resistance mutations to dolutegravir.

The list of mutations can be found in Table 1.

**Table 1:** Mutations data.

ID	Subtype	Accessory mutations	Other mutations
002	B		E11D, S17SN, A21T, L45LQ, M50MI, D64DN, S119T, T124TA, V201I, T206TS, L234I, D256E, I268L, D270H, S283G, R284G
004	F		E11D, S24N, V31VI, V37I, I72V, S119P, T122I, T125A, I135V, M154I, V165I, V201I, K211R, T218TS, L234V, D256E, S283G
005	F		E11D, S24N, V37I, M50I, I72V, S119P, T122I, T125A, I135V, M154I, V165I, V201I, K211R, L234V, D256E, S283G
006	B		D6T, K7E, E10D, S119T, T124N, T125A, V201I, T206S, S230N, A265V, I267M, D286N
008	B		E11ED, K14KR, V32I, I72V, K111KE, S119T, V201I, S230N, R231K
010	B		E11D, M50I, I60V, S119P, T124A, D167E, S195T, A205S, D207E, I217V, S230N, L234I
012	B		E11D, K14KR, L28I, V32I, A91AITV, L101I, T112V, I113V, T124N, T125A, F181L, T218I, N222K, S255Q
013	B		K14R, V31I, S39SN, I72V, S119P, T124TA, T125A, I135V, E157EA, L158LI, V201I, A205S, I220L, Q221S, N222K, L234LI, R269K
014	B		E11D, R20K, L101I, S119SG, T122TI, D167E, G193D, T218S, S255G, D256E, I267V, I268L, S283G, D288N
017	F		E11D, D25E, V31I, V37I, I72V, I84L, S119P, T122I, T124A, T125A, G134N, K136Q, V201I, T218I, L234V, D256E, S283G
018	B		K14KR, S17SN, I72IV, P90PS, L101LI, T124NS, K136KN, K160T, T206S, L234I, S283G
019	B		S17N, V31I, V32I, D41DN, M50I, G59E, I60M, T124A, T125A, K156N, V201I, D207N
021	B		D6E, E11D, S17C, I72V, L101LIM, I113V, S119P, T122TI, T125A, K188KR, V201VI, T206TS, A265V, R269RK, S283D, D286N
024	F		E11D, V31I, V32I, V37I, S39N, L45Q, I72V, A98G, L101I, S119P, T124N, T125TA, K136Q, V165I, V201I, K211R, T218I, L234V, D270N, S283G
025	F		E11D, V31I, V32I, V37I, S39N, K42R, L45Q, A98G, L101I, S119P, T124N, T125A, K136Q, V165I, V201I, K211R, T218I, L234V, D270N, S283G
028	B		V31I, S39N, A91S, L101I, T112A, T125TA, D167E, V201I, T218S, D253E, N254D, S255R, M275V
029	B		S24SN, V88I, T124N, T125A, V126L, G163E, T206S, R284G, D286N
030	B		E11D, K14R, S24N, V31I, V32VI, I72V, S119G, T122I, T125A, V201I, T206S
031	B		E10A, E11D, S17N, D25E, S39C, L45V, I60V, L101I, I113V, S119T, T124N, T125A, G189GR, V201I, K211T, Q216H, I220N, S230N, D253E, N254D, D256E, S283G
033	C		V31I, I72V, T112V, S119T, T124N, K136Q, V201I, T206S, L234I, I251L, D256E, A265V, R269K, D278A, V281M
035	B		V31I, M50I, L101I, T122I, T125A, V151I, V201I, A265V
036	C		E11D, M22L, D25E, V31I, V37I, D41N, M50I, I60V, E96D, L101I, G106A, T112V, S119R, T122S, T124N, K136Q, D167E, T218L, L234I, I251L, R269K, D278A, V281M, S283G
037	C		E11D, M22L, D25E, V31I, V37I, D41N, M50I, I60V, E96D, L101I, G106A, T112V, S119R, T122S, T124N, K136Q, D167E, T218L, L234I, I251L, R269K, D278A, V281M, S283G
038	B		E11D, M22L, D25E, V31I, V37I, D41N, M50I, I60V, E96D, L101I, G106A, T112V, S119R, T122S, T124N, K136Q, D167E, T218L, L234I, I251L, R269K, D278A, V281M, S283G, P30S, I72V, L101I, K103KR, S119P, T122I, T124A, I182V, G193E, T206S, I208L, T218L, L234I, D279G
039	B		V31I, S119P, T122I, S195T, T206S
044	B		K7KR, V77VA, T124N, V201I, S230N, A265V, S283G
048	B		S17N, M22L, L28M, E35Q, D41N, M50I, K103R, T124N, T125A, V126M, G163E, T210I, Q216H, D286AT
049	F		P30PS, V32I, G106A, S119P, T124TA, K136Q, V165I, V201I, L234I, D256E
053	B		D25E, V31I, I72IV, I113V, M178MV, K211RT, L234I, N254S, S255G, A265V
058	B		D6E, E11D, S17N, S39N, L45I, I72V, L101I, S119P, T124N, T125M, I135V, K156N, D167E, V201I, K211R, Q221K, N222K, S230N, V260I
059	C		E11D, D25E, V31I, L45I, M50T, L101I, T112V, S119P, T124N, K136Q, M154MV, D167E, V201I, T206S, K211KN, L213LQ, T218I, L234I, I251L, D256E, A265V, R269K, D278A
060	B		K14KR, S24N, V31VI, M50I, V54VI, G59GE, G70GD, L74I, I113V, T124A, T125A, V151VI, K160KN, D167E, V201VI, T206S
063	B		E11D, S24N, V31I, L101I, T122I, T124N, P142PS, D253E, D256E
065	C		E11A, V31I, A38AT, L45V, M50I, L101I, K111R, T112V, T124N, K136Q, G163E, K188R, V201I, K211R, T218I, L234I, I251L, S255G, A265V, D278A, V281M, R284G, D286N
069	B		D6E, E10D, V31I, L45I, K71N, I72V, A98G, L101I, S119R, T124N, K156N, D256E
072	B		D6E, E11D, S17C, I72V, L101I, S119P, T125A, G193E, S283G, D286N
073	B		L63I, A91AS, L101I, S119P, L234LV, V249VI, D256E, S283G
075	B	T97A	E11ED, V31I, L101I, S119P, K136N, V201I, T206S, I208IM, T210S, I220L, Q221H, D288G
078	C		E11D, E13D, M22L, D25E, V31I, V37I, M50I, I60V, P90T, L101I, K103KN, G106A, K111KN, T112V, S119R, T122S, T124N, K136Q, D167E, K188R, V201I, T218L, L234I, I251L, D256E, R269K, D278A, V281M, S283G
082	B		E11D, S17T, A23V, L28I, P30A, V37I, I60M, L101I, K103R, T125A, K156N, D167E, I203M, K215N, Y227F, D253H, D256E, S283G
081	F		E11D, I84L, L101I, S119P, T122I, T125A, K136Q, D167E, V201I, L234V, D256E, S283G
083	B		S17N, I72V, T112I, S119P, T124A, T125A, K136R, V201I, D256E
084	B		E11D, S17N, L28I, L101I, K111R, S119GR, T125A, K156N, H171Q, L234I
085	CRF47_BF		S17N, I60IM, G70GE, I72V, I113V, E152EK, M154I, V165VI, R231RG, L234V, D256E, S283G
087	CRF47_BF		E10D, M50T, I72V, L74I, V165VI, K173R, G193E, L234V, D256E, S283G
090	B		K7R, S39C, L101I, G163E, V201I
092	C		K14KR, S24SN, E35EK, G47GR, M50T, L101I, T112V, T124N, K136Q, V201I, I208IM, L234I, V281M
094	B		E11D, R20K, D41N, I113IV, S119SAGT, T122TI, T125A, V151I, L234V, D253E, S283G
095	F		K14R, S17N, V31VI, V32I, L45V, I72V, I84L, L101I, K111R, S119P, T124A, T125A, K136Q, Q177L, V201I, T218I, L234V, D256E, S283G
097	B		E11D, S24N, V31I, T112I, I113V, T124N, T125A, V151I, V201I, N254Q
098	B		E11ED, K14KR, L28I, I60V, K111Q, I113V, T124N, D167E, I208L, S230N, D256E
099	B		E11D, A23AV, D25E, A38S, S39C, L45Q, A49P, M50I, L74I, L101I, K103R, I113V, T124A, T125A, D167E, H171Y, L234I, D253E, N254K
100	B		E11D, A21T, E35EQ, S39SN, L45V, M50I, V151I, G163A, I208M, N254Q, D256E
101	B		D3E, D6N, K7E, V31I, L101I, V151I, V201I, K211R, T218I
103	B		S17N, L68V, I72V, I73V, A91S, L101I, T125A, I135V, K156N, I203IM, T218I
104	B		E10D, L101I, S119T, T125A, K156N, S230N, D256E
106	B		L101I, I182V, V201I, D256E, A265AV, I268IL
107	B		E11D, V31I, V32VI, M50I, I72V, L101I, S119P, T122I, T124A, F181L, I182V, V201I, T206S, D256E
109	B		S17N, V31I, L45LI, K111KR, T124N, T125A, K156N, V201I, A265AV
110	B		S17N, I72V, L101I, T112I, T124A, V151I
111	B		S17N, A23AS, L28I, I72V, T124A, T125A, V126L, A265V
112	B		E11D, R20K, L28I, V37I, S39C, I60M, I72IV, V77A, L101I, T124S, T125A, M154L, V165I, G193E, D207N, K211T, D279N, S283G
117	B		E11D, R20K, L28I, T124A, D167E, V201I, K215N, T218I, D256E, S283G
118	B		K7KQ, E11D, A23V, D25E, V31I, L101I, T112I, I113V, V201I, L234I
120	B		T124N, M154I, G163S, V201I, T218I, Q221KN, P233PS, L234V
122	C		E11D, S24N, D25E, V31I, M50T, L101I, T112V, T124N, I135V, K136Q, V201I, T218I, L234I, I251L, A265V, R269K, D278A, V281M
123	C		V31I, M50I, L101I, T112V, S119P, T124N, G134D, K136Q, K188KR, V201I, T218I, L234I, I251L, A265V, R269K, D278A, S283G
124	B		S17N, R20K, V31I, V37VI, M50MI, I72V, I84L, A91S, L101I, K111KR, S119P, T124A, I135IV, K136NT, V165I, V201I, K211R, T218I, L234V, D256E, A265V, S283G
125	B		S17N, L28I, I72IV, V77A, V88VI, T125A, K156N, V201I, I208L, K219N, N222K, S230N, V249VL, D256DA, S283G
127	B		D6NS, K7E, E11A, S17SN, A21AS, L28I, M50I, V54I, I72V, I84IM, G106A, T112A, S119P, T122I, T125A, V201I, T206TS, L234I, D279DH, V281M
128	B		D6N, K7E, E11A, L28I, M50I, V54I, I72V, G106A, T112A, S119P, T124N, T125M, V201I, L234I, V281M
131	B		S17N, S39C, L101I, D167E, V201I, Q216H, Q221QKR, D256E, V281M
133	B		E11D, D25E, L74I, I84L, L101I, K111Q, T112V, D167E, V201I, A205S, T206S, D256E
134	B		L101I, I182V, G193E, V201I, D256E, D278N
135	B		K14R, S17N, S119R, T125A, G163E, Q216H, T218E, D278E
136	B		K14KR, S17N, V31VI, S39C, L101I, T124NS, T125A, A129S, K136N, V201I, V281M
137	B		E11ED, S17SN, L28I, V37VI, L45I, I84IM, L101I, K160N, V201I, D232DE, D256E
138	B		E11D, R20K, D25E, V31I, L101I, T122I, D256E, D286N
140	B		M50I, I72V, L101LI, K111T, T122I, T206S, P261T
144	B		K14R, A21AT, I60IV, L63M, T125V, I208L, P261PT
145	B		S17N, I72V, L101V, S119P, T122I, T124N, V201I
147	B		D6S, K7E, E11D, F26Y, V31I, V37I, M50I, L63I, L101I, I113V, T122TI, T124TA, T125A, K127KR, N254Q, S255G
148	B		D6E, S17N, L28I, P30A, L101I, T124A, G163Q, V201VI, K240R, D256E
149	B		K7R, S17N, L45I, M50MT, L101I, T112TIMR, T124A, K160N, D167E, V201I, I220L, Q221S, D256E, S283G
150	B		D6T, K7E, E10D, V31I, M50I, T125A, V201I, Q216H, S230N, R231K, K258KEGR, A276AT

## Discussion

Primary resistance to DTG, commonly used in HIV treatment, has been a subject of increasing concern, especially as its use has become more widespread. Surveys reported that levels of resistance to dolutegravir ranged from 3.9 to 8.6 % and reached 19.6 % among people experienced with treatment and transitioned to a DTG-containing ART while having high HIV viral loads.^10^, ^11^, ^12^

A study published in 2014 demonstrated that subtype B had a prevalence of approximately 70 % among individuals, with the presence of subtype F and BF recombinant forms accounting for around 20 %.^13^ By 2019, however, there appears to be a distribution shift with the inclusion of subtype C and BC recombinants, primarily in the southern part of the country followed by the central-west region.^14^ This coincides with our sampling.

The DTG RESIST study^15^ utilized data from various HIV cohorts to analyze the patterns of Drug Resistance Mutations (DRMs) and to identify the risk factors associated with resistance to dolutegravir. They included 599 individuals who underwent genotypic resistance testing on dolutegravir-based ART between May 22, 2013, and December 20, 2021. Most had HIV-1 subtype B (*n* = 351, 59 %), a third had been exposed to first-generation INSTIs (*n* = 193, 32 %), 70 (12 %) were on dolutegravir dual therapy, and 18 (3 %) were on dolutegravir monotherapy. INSTI DRMs were detected in 86 (14 %) individuals; 20 (3 %) had more than one mutation. Most (*n* = 563, 94 %) were susceptible to dolutegravir, 7 (1 %) had potential low-level resistance, 6 (1 %) had low-level resistance, 17 (3 %) had intermediate-level resistance, and 6 (1 %) had high-level dolutegravir resistance. The risk of dolutegravir resistance was higher on dolutegravir monotherapy and dolutegravir plus lamivudine dual therapy.

In our study, there was no primary or long-term resistance in the group that maintained the triple combination or in the group that switched to the dual combination. Over the 48-weeks of follow-up, we did not observe an increase in viral load that would allow us to perform a new genotyping.

Diaz et al. studied sequences generated from the plasma of 113 patients with confirmed virologic failure to first-line 3TC/TDF plus DTG treatment in the Brazilian public health system before December 31, 2018. Major Integrase Resistance Associated Mutations (INRAMs) were detected in seven patients (6.19 %). Mutations not related to 3TC/TDF plus DTG, and therefore likely representing Transmitted Drug Resistance (TDR), were found in 28 patients (24.8 %). Twenty-five patients (22.1 %) had mutations associated with nucleoside reverse transcriptase inhibitors, and 19 patients (16.8 %) had mutations associated with non-nucleoside reverse transcriptase inhibitors.^16^ We did not find mutations conferring transmitted resistance to Nucleoside Reverse Transcriptase Inhibitors (NRTIs) or Non-Nucleoside Reverse Transcriptase Inhibitors (NNRTIs), and the few individuals who experienced viral load escapes were counseled on the importance of adherence to treatment. The accessory mutation T97A, which we identified, is a prevalent polymorphic mutation associated with resistance to INSTIs. Its prevalence ranges from 1 to 5 % among INSTI-naïve individuals, varying by subtype. This mutation is selected by each of the INSTIs. Individually, T97A decreases Elvitegravir (EVG) susceptibility by approximately 3-fold, while having minimal to no impact on other INSTIs. However, when combined with other INSTI-resistance mutations, T97A significantly reduces susceptibility to all INSTIs.^17^

There are some limitations to consider in this study. Firstly, the sample size was relatively small and sourced from a single reference center, which may restrict the generalizability of the results to broader and more diverse populations. Secondly, genotypic resistance testing was only feasible for a subset of participants due to issues such as low viral loads or amplification failures, which may have resulted in missed resistance profiles. Additionally, challenges related to treatment adherence and follow-up compliance led to 19 participants being lost to follow-up, potentially impacting the robustness of the findings. These limitations highlight the necessity for future multi-center studies with larger and more representative cohorts to confirm and extend these observations.

Despite these limitations, this study provides compelling evidence supporting the use of DTG as a cornerstone of first-line HIV treatment in Brazil. The findings suggest that DTG-containing regimens are highly effective, well-tolerated, and maintain a substantial barrier to resistance, solidifying their role as a critical component of the national HIV treatment strategy. Nonetheless, close monitoring of adherence to antiretroviral therapy remains essential to ensure optimal outcomes.

## Conflicts of interest

The authors declare no have conflicts of interest.
